# Perceived stigma, discrimination and psychological problems among patients with epilepsy

**DOI:** 10.3389/fpsyt.2022.1000870

**Published:** 2022-11-09

**Authors:** Najma Iqbal Malik, Rabia Fatima, Irfan Ullah, Mohsin Atta, Ashraf Awan, Abdulqadir J. Nashwan, Saeed Ahmed

**Affiliations:** ^1^Department of Psychology, University of Sargodha, Sargodha, Pakistan; ^2^Kabir Medical College, Gandhara University, Peshawar, Pakistan; ^3^Rai Medical College, Sargodha, Pakistan; ^4^Hamad Medical Corporation, Doha, Qatar; ^5^Rutland Regional Medical Center, Rutland, VT, United States

**Keywords:** epilepsy, perceived stigma, discrimination, psychological problems, anxiety

## Abstract

**Objective:**

The current study sought to investigate the effects of perceived stigma and discrimination on epilepsy patients' psychological problems, such as depression, anxiety, and quality of life.

**Study design:**

A purposive sampling technique was used in this cross-sectional study.

**Materials and methods:**

The sample consisted of 186 patients with epilepsy. The Stigma Scale, Depression Anxiety Stress Scale, and Quality of life in Epilepsy-10 were used to measure the study variables.

**Results:**

Findings showed that both the perception of stigma and discrimination have a significant positive correlation with depression as well as anxiety. Stigma is a significant positive predictor of depression and anxiety at [F (1, 184) = 27.8, 15.92, *p* < 0.001, 0.001, respectively] and explains 12.7 and 7.5% variance that could be attributed to Stigma. Stigma is also a significant predictor of quality of life at [F (1, 184) = 16.10, *p* < 0.001] and explains the 7.5% variance that could be attributed to Stigma. Results also indicate that discrimination is a significant positive predictor of depression and anxiety at [F (1, 184) = 32.39, 19.91, *p* < 0.001] and explains 15 and 9.8 % variance, respectively, that could be attributed to Stigma. However, stigma negatively predicts quality of life at [F (1, 184) = 20.34, *p* < 0.001] and explains 10 % variance. Non-significant differences were found in all the demographic variables (i.e., gender, socio-economic status, and disease duration), except the quality of life was significantly high among individuals with high Socio-economic status.

**Conclusions:**

Stigma is significantly higher in epileptic patients and has a detrimental effect on the patient's quality of life, recovery, and prognosis. Thus, there is undoubtedly a need to address psychological issues, most notably the stigma associated with illnesses. Psychologists, psychiatrists, other physicians, and care givers of epileptic patients must pay close attention to the stigma in this patient population.

## Introduction

Individuals suffering from psychiatric conditions face enormous challenges in their daily lives. People with severe mental illnesses are more likely to have low self-esteem, internalization of negative beliefs, a low level of hope, difficulties with social relationships, a lower likelihood of adhering to treatment, difficulties at work, unemployment, and all of this is driven by stigma and discrimination, which eventually has a negative impact on recovery. In many ways, they are marginalized and discriminated against and are not given the opportunities that define a good life. Many people with severe mental illnesses struggle to meet basic needs such as education, safe and stable housing, good jobs, and decent healthcare. In addition to mental illness, stigmas surround neurological disorders such as epilepsy. Despite medical advances, there are still stigmas and misunderstandings about epilepsy. The stigma associated with epilepsy includes both the stigma experienced by epilepsy patients and the community's attitudes and beliefs about them. Patients with epilepsy may feel ashamed and embarrassed if they have a seizure in public due to symptoms such as limb shaking, staring spells, chewing, and urinary/bowel incontinence (1). Unfortunately, they face prejudice and stigma from others as well because they are perceived as insane, possessed by evil spirits, and having weak minds. The word “epilepsy” conjures up images of a person having fits (seizures) at any time, which has a negative impact on their social relations and quality of life. People with epilepsy (PWE) who live in underprivileged areas, unfortunately, do not receive appropriate care, which often results in their illness remaining untreated, affecting both their physical and psychological health (2).

Perceived stigma is a risk factor for the development of psychiatric conditions such as anxiety and depression in people with epilepsy. According to a recent study, patients with epilepsy perceived 25% of high stigma 55.0% anxiety, and 47.5% severe depression (3). Epilepsy-related stigma is not only prevalent in developing European and Middle Eastern countries but also in developed countries (4, 5). Even though physicians are meticulous in diagnosing and treating epilepsy, they invariably neglect to address the associated stigma, which can lead to psychological problems such as depression, anxiety, and suicidality (6). Psychological morbidity is significantly more prevalent in people with epilepsy than those without the condition (7). The psychological morbidity includes increased levels of depression and anxiety, social isolation, and withdrawal (8). People having epilepsy face several social problems and these social aspects have more negative effects as compared to the disease itself. Studies revealed that individuals with epilepsy are likely to have worse self-worth and have a high rank of depression and anxiety than individuals without epilepsy (9).

Individuals experiencing epilepsy suffer from depression at two to three times the rate of the general population (10), and they are more likely to suffer from depression than people with other chronic conditions (11). Studies have found that there is a substantial connection between depression, anxiety disorders, and epilepsy. Approximately one in five of all adults who experience epilepsy suffer generalized anxiety disorder (GAD), and at times epileptic children show symptoms of depression and anxiety (12–14).

Quality of life also has an impact on the people living with epilepsy, and empirical evidence revealed that the individuals with epilepsy as a cluster have poor QOL as compared to those without epilepsy (15, 16). For example, Yeni and his colleagues (17) conducted a study in which they compared health-related QOL in adults with and without seizures in a large sample and reported that patients with seizures reported high levels of psychological distress, sleep problem, and pain as compared to the individuals without seizures and were also more likely to develop serious mental illnesses.

Researchers have made significant progress in gaining a better understanding of the neurobiological basis, diagnosis, and treatment of epilepsy. However, psychological problems persist and have a detrimental effect on recovery from this condition and prognosis. Thus, there is certainly a need to resolve psychological issues, particularly the stigma associated with illnesses. As most of the previously done studies have been conducted by physicians and neurologists, so they lack prevalence data (18), frequency, mode of onset, and causes of epilepsy (19). Psychologists and psychiatrists need to focus on the psychological as well as social issues of people with epilepsy. According to World Health Organization (WHO), people with epilepsy can live a normal life medically, but unfortunately, this stigma related to the disease affects their lives; even having no medical difficulties makes their life more hard (20).

According to WHO around 50 million people worldwide suffering from epilepsy, in developing countries, almost 80% of people live with epilepsy. There is 3 times more premature death for normal people with risk of epilepsy. 3 quarters of people with epilepsy living in developing countries do not get the treatment they need. The global prevalence of epilepsy is generally taken as between 5 and 10 cases per 1000 persons by WHO in 2019. Few studies have come from developing countries (21). Few epidemiological studies of epilepsy are available from Pakistan (22). Epilepsy has not been thoroughly investigated in Pakistan and epilepsy has a huge prevalence rate of 9.98% per 1,000 populations, in Pakistan that is twice as common in rural areas (23) mostly among younger population less 30 years of age. It has been estimated that its spread rate is greater in countryside areas than urban areas. About 25% cases are genetic and different types of seizure are present in developing countries (24). So, Family history is an important risk factor for this disorder. This prevalence of epilepsy and their psychosocial issues were also found to be dependent upon the country's health care system, socioeconomic status and community response toward patients. Especially in collectivist country like Pakistan it is very much evident that psychological health of epileptic patients and even their caregivers have been strongly associated with the attitudes and perceptions (stigma and discrimination) of their community as well which are more harmful than the disease itself (25).

Epilepsy patients' demographics have also been studied in Pakistan, (26) but very few studies are conducted on the psycho-social factors affecting the patients with epilepsy in Asian countries (27–34). Given the scarcity of studies in the region investigating psychological problems in epilepsy patients, this study was conducted to investigate the effects of perceived stigma and discrimination on individuals with epilepsy, with a focus on depression, anxiety, and quality of life variables.

## Materials and methods

In the present study, a co-relational research method was used, and a sample of 184 individuals with epilepsy was collected by using a purposive sampling technique. Both genders were included in the sample, i.e., 107 males and 79 females. 92 (49.5 %) were married and 94 (50.5%) were unmarried. Their education level was primary to bachelors and were able to read and write easily. 36.6% individuals were suffering from this disease from 1 to 10 years, 32.3% had the disease from 11 to 20 years, 28.5% had this from 21 to 30 years and in 16.1% suffering duration was from 31 to 40 years. 52.2% patients were only one epileptic in their family, 25.3% had two patients in their family, and 22.6% had three epileptic patients in their family. 43% had low socioeconomic level (5,000/−30,000/- PKR), 30.1% had moderate level (31,000/−80,000/ PKR) and 26.9% had high socio economic level (81,000/- PKR and above)in accordance to Pakistan economic survey of 2020.

Data for the present study was collected from several cities of Punjab, i.e., Brig. Rashid Qayyum's Psychiatric Clinic C.M.H Sialkot, Col. Sajjad Psychiatric Clinic Attock, and Awan Clinic Sargodha. As an inclusion criterion the patients who were diagnosed having four main types of epilepsy: focal, generalized, combination focal and generalized, and unknown with history of multiple seizure attacks patients were part of study. The patients of young and middle adulthood (19–65 years) were included in sample (Erickson, 1950). However, the patients who had only one seizure attack history were not made part of sample. The people with epilepsy with age less than 18 or above 65 were also excluded from the study.

The study use 28 itemstotal scores on stigma scaleto measure the constructs of stigma whereas one of its sub-scalenamely discrimination was used to measure the variable of discrimination in present study. The stigma scale is originally comprised of 3 sub-scales (viz. discrimination, disclosure, and positive aspects), of which nine items were reversely coded. It is a self-report measure with five-point Likert-type response format. The Cronbach's alpha for the stigma scale was 0.87, and the subscale discrimination, disclosure, and positive aspects were found to have 0.87, 0.85, and 0.64, respectively (35). Depression Anxiety Stress Scale (36) and Quality of life in Epilepsy-10 (37) were used to measure the psychological problems. Depression anxiety stress scale consists of 21 items and has three subscales, depression (item no. 3, 5, 10, 13, 16, 17, and 21), anxiety (item no. 2, 4, 7, 9, 15, 19, and 20) and stress (item no. 1, 6, 8, 11, 12, 14, and 18). In the present study, Depression and Anxiety subscale was used. Quality of life in epilepsy scale consists of 10 items and has six subscales, i.e., emotional well-being (1, 2), social functioning (3), overall quality of life (4), cognitive functioning (5, 9), seizure worry (6–8) and medication effects (10).

Participants were only recruited in the present study if they fulfilled the criteria. According to the criteria, individuals who were diagnosed with epilepsy at least a year ago were eligible to participate. After the recruitment of participants, their consent to take part in the present study was obtained. The authorities of the institutes were also approached, and their policies were taken into consideration. The researcher personally administered the questionnaires to the participants to avoid any errors. Furthermore, the obtained data were analyzed using SPSS version 22, and to test the hypothesis of the present study; regression analysis was applied.

### Statistical analyses plan

In order to accomplish the objectives of present study various statistical analyses were executed. First of all, data was subject to descriptive analysis for screening purpose then kurtosis and skewness analyses were used to scan the normalcy of data for further processing of data. Reliability coefficients were computed the psychometric soundness of measurement instruments. Pearson correlation was applied to peep into the initial relationship pattern among variables then regression analysis was accounted for determining causal relationship. Tables in following section elucidate the statistical findings of current study.

## Results

The Table 1 shows the means, standard deviations, and alpha reliabilities as found in the present research, which ranges from 0.70 to 0.89 and it is considered satisfactory. The table also shows Pearson correlations between the study variables.

**Table 1:** Psychometric properties and Pearson correlation among variables.

**Variable**	**1**	**2**	**3**	**4**	**5**	***M***	***SD***	**α**	**Range**		
									**Potential**	**Actual**	**Skewness**
1	–	0.98^***^	0.36^***^	0.28^***^	– 0.28^***^	37.27	6.56	0.73	11–55	21–54	0.23
2		–	0.38^***^	0.31^***^	– 0.32^***^	91.55	15.41	0.86	28–140	53–129	0.33
3			–	0.89^***^	– 0.46^***^	21.15	5.03	0.89	7–28	7–28	– 1.08
4				–	– 0.45^***^	21.10	4.87	0.89	7–28	7–28	– 0.99
5					–	38.39	6.11	0.70	10–51	22–51	– 0.10

1, perceived stigma; 2, discrimination; 3, depression; 4, anxiety; 5, depression anxiety total; 6, quality of life.

*** *p* < 0.001.

**Figure 1:**
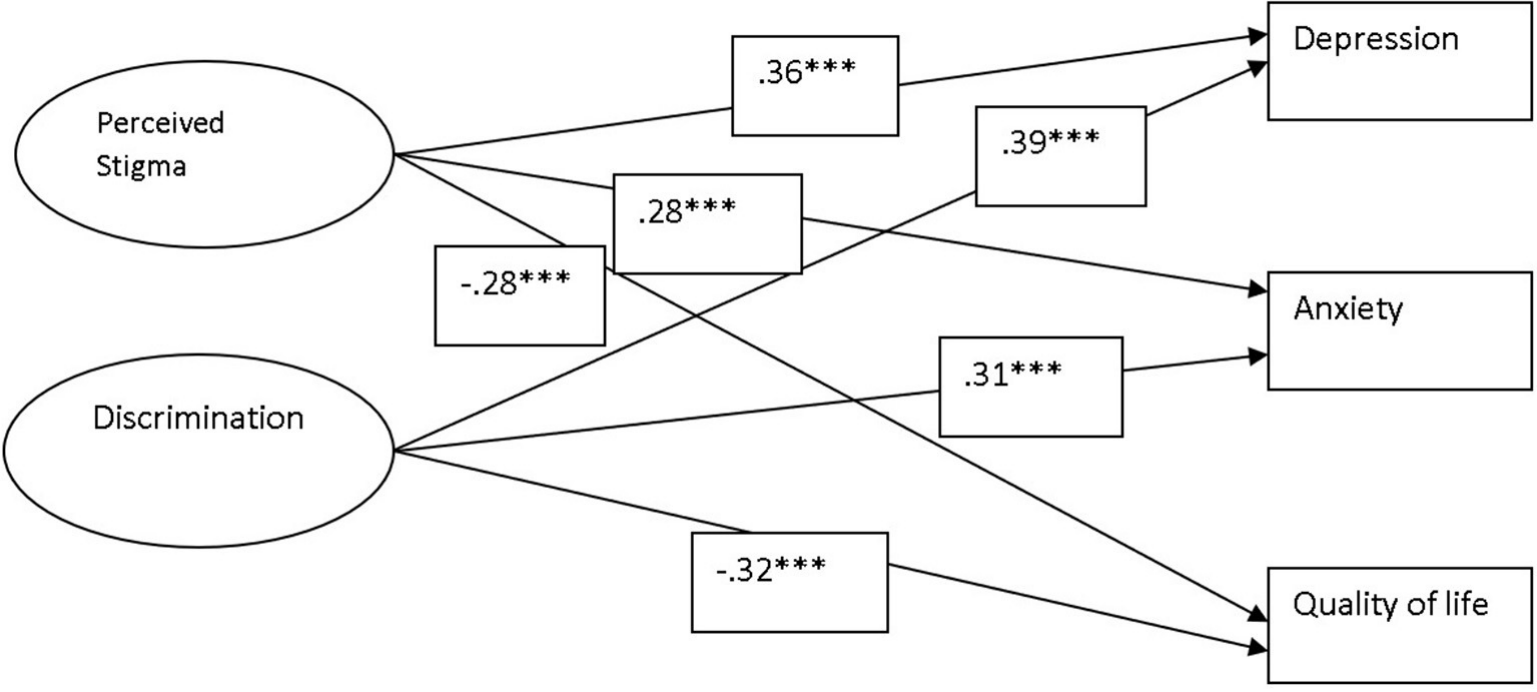
Outcome model of study. ^***^p < 0.001.

The result in Table 2 shows that stigma as significant positive predictor may increase the depression and anxiety at [*F* (1, 184) = 27.8, 15.92, *p* < 0.001, 0.001, respectively] among people with epilepsy and this increase is assumed to be the 12.7 and 7.5% in both respectively. Stigma is also a significant negative predictor of quality of life at [*F* (1, 184) = 16.10, *p* < 0.001] thus may decrease almost 7.5% of the quality of life among people with epilepsy. Results also indicate that discrimination as significant positive predictor may cause 15 and 9.8% increase in depression and anxiety [*F* (1, 184) = 32.39, 19.91, *p* < 0.001] accordingly. However, stigma negatively predicts quality of life at [*F* (1, 184) = 20.34, *p* < 0.001] and explains 10% variance. Same are explicitly evident in Figure 1.

**Table 2:** Linear regression showing the effect of perceived stigma and discrimination on depression, anxiety, and quality of life.

**Variables**	**DEP**		**ANX**		**QOL**	
	***R* ^2^**	**β**	****R* ^2^***	**β**	***R* ^2^**	**β**
PS	0.127	0.363^***^	0.075	0.282^***^	0.075	– 0.284^***^
Disc	0.150	0.387^***^	0.098	0.313^***^	0.100	– 0.316^***^

PS, perceived stigma; Disc, discrimination; DEP, depression; ANX, anxiety; QOL, quality of life.

*** *p* < 0.001.

Results in Table 3 show that there are no significant gender differences in the present sample.

**Table 3:** Independent samples *t*-test showing mean, standard deviation and *t*-values for gender among study variables.

**Scales and Subscales**	**Male (*****n*** = **107)**		**Female (*****n*** = **79)**			**95% CI**			
	***M***	***SD***	***M***	***SD***	***t* (184)**	***p***	***LL***	***UL***	***Cohen's d***
PS	90.42	15.42	93.09	15.36	– 1.16	0.24	– 7.17	1.83	– 0.17
Disc	36.79	6.61	37.94	6.48	– 1.18	0.23	– 3.07	0.768	– 0.17
DEP	21.42	4.87	20.78	5.25	0.86	0.38	– 0.83	2.12	0.12
ANX	21.41	4.53	20.69	5.30	0.98	0.32	– 0.71	2.14	0.14
QOL	38.94	5.86	37.64	6.40	1.43	0.15	– 0.48	3.08	0.21

PS, perceived stigma; Disc, discrimination; DEP, depression; ANX, anxiety; QOL, quality of life.

The result in Table 4 shows the mean, standard deviation, and *F* values for socioeconomic status on perceived stigma, discrimination, depression, anxiety, and quality of life. Findings indicate that there are no differences in all study variables except the quality of life, which indicates that patients with epilepsy belonging to high socioeconomic status have a high quality of life as compared to the other groups.

**Table 4:** ANOVA analysis showing mean, standard deviation and *F* value for socioeconomic status among epilepsy patients on variables of the study.

	**Low SES** **(*****n*** = **80)**		**Moderate SES** **(*****n*** = **56)**		**High SES** **(*****n*** = **50)**				
**Variables**	***M***	***SD***	***M***	***SD***	***M***	***SD***	***F***	***p***	***Post-hoc***
PS	91.63	14.06	90.82	17.43	92.26	15.34	0.11	0.89	
Disc	37.29	5.90	36.89	7.51	37.68	6.54	0.18	0.82	–
DEP	21.36	4.34	20.25	6.09	21.84	4.71	1.44	0.23	–
ANX	21.03	4.63	20.58	5.64	21.80	4.32	0.82	0.43	–
QOL	36.82	6.46	39.42	6.54	39.74	4.32	4.83	0.01	1 < 2 < 3

PS, perceived stigma; Disc, discrimination; DEP, depression; ANX, anxiety; QOL, quality of life.

The result in Table 5 shows no significant differences in terms of duration of disease, among patients with epilepsy, on perceived stigma, discrimination, depression, anxiety, and quality of life.

**Table 5:** ANOVA analysis showing mean, standard deviation and *F* value for duration of disease among epilepsy patients on variables of the study.

	**1–10 years (*****n*** = **68)**		**11–20 years (*****n*** = **60)**		**21–30 years (*****n*** = **35)**		**31–40 years (*****n*** = **23)**			
**Variables**	***M***	***SD***	***M***	***SD***	***M***	***SD***	***M***	***SD***	***F***	***p***
PS	91.65	14.59	92.65	15.08	87.69	17.28	94.30	15.55	1.08	0.35
Discrimination	37.49	6.31	37.75	6.37	35.23	7.10	38.52	6.68	1.55	0.20
Depression	20.80	5.16	21.70	4.86	20.34	5.42	22.00	4.46	0.85	0.46
Anxiety	20.75	4.96	21.86	4.66	20.20	5.42	21.56	4.20	1.07	0.35
QOL	38.64	6.09	38.61	5.28	38.05	7.00	37.56	7.04	0.23	0.86

PS, perceived stigma; Disc, discrimination; DEP, depression; ANX, anxiety; QOL, quality of life.

## Discussion

According to the WHO reports (2022), 49 per 100,000 people are diagnosed with epilepsy in high-income countries each year; however, this figure is quite high in low-income countries, i.e., 139 per 100,000 each year (38). Individuals with epilepsy face a variety of psychosocial issues in addition to physical hazards. Labeling someone with epilepsy itself has its toll, and it comes with a stigma attached to it (39). Thus, the current study is an attempt to shed light on some of the psychosocial difficulties that people with epilepsy face in Pakistan. The findings of this study were similar to previous studies in that stigma perception has a significant positive correlation with depression. The previous studies showed similar results, e.g., Yildirim et al. (9) also worked on epilepsy patients of age ranging from 19 to 65 and found a significant positive correlation (*r* = 0.30, *p* < 0.001) between stigma and depression. Souza et al. (5) studied sixty epilepsy outpatients of a hospital in Campinas, Brazil, with ages ranging from 20 to 45 years and found 26.6 percent perception of stigma and 31.6 percent depression in the epilepsy patients. Another research study also found similar results, i.e., Walsh et al. (11) studied the perceived psychosocial consequences of epilepsy and found that the stigma perceptions are related to increased psychosocial health consequences such as depression and anxiety in epileptic patients.

Wang and his colleagues (8) conducted a study on 300 patients, both male and female. The research found that epilepsy stigma had a significant impact on depression and quality of life. In addition, Blaszczyk et al. (13) investigated the role of social support in epilepsy self-help groups. Researchers measured the level of stigma, anxiety, and depression in their study, and stigma was found to be significantly correlated with anxiety and depression. Similarly, the current study hypothesized that a significant positive correlation exists between perceived stigma and anxiety, which is consistent with previous studies such as Oluwole, Obadeji, and Dada (12), which investigated the determinants of anxiety and depression in patients with refractory epilepsy and found 40.63% depression and 71.43% anxiety in them. Depression and anxiety both showed a positive correlation with stigma in their study. Besides that, the research found that 42.9% of epileptic patients with depression felt stigma, while 71.4% of epileptic patients with comorbid anxiety felt stigma. According to de Souza et al. (5) people with epilepsy attach a stigma to themselves or their epilepsy, resulting in discrimination, which causes epileptics to be preoccupied with their disease and spend much of their psychological energy on disclosure anxiety vigilance, and a consequent uncertainty of identity. In a study conducted in Brazil, Souza et al. (5), sought to examine anxiety and depression in patients with epilepsy and their relationships to psychological and neuro-epilepsy variables. They noticed that 33.3% of epilepsy patients suffered from anxiety. Additionally, epilepsy was associated with the disease (63.4%), mental distress (11.6%), feelings of dishonor, fear, agony, low self-esteem (56.6%), and the perception of stigma (26.6 %).

Perceived stigma intensifies stressful situations and impairs the patient's ability to cope with them. As a result, many people with epilepsy experience anxiety when interacting with society. The fact that anxiety appears to be strongly correlated with stigma explains why mainstream reports have found elevated anxiety levels among people with epilepsy in varied countries (14). Present study hypothesized that an inverse relationship between quality of life and perceived stigma exists among epilepsy patients, which was supported by the findings in the present study. Another study examined the impact of epilepsy on the health-related quality of life (HRQoL) of Asian adults with epilepsy, as well as the associated factors. Thirty-six articles were reviewed for this reason. The HRQoL of Asian adults with epilepsy was reported to be lower than that of the people without the condition. (14) Epilepsy affects both mental and physical health. Many Asian countries had negative attitudes and stigmas toward persons with epilepsy in employment and marriage (5, 40, 41).

Similarly, Singh and Pandey (34) studied 64 older adults and found that their health-related quality of life scores was significantly lower (HRQoL). Their research also found a link between high perceived stigma and frequent seizures and poor psychosocial function and quality of life. Other studies' findings mirrored the present study, e.g., In a comparative study, Vaingankar et al. (10) found that people with epilepsy have a lower quality of life than the general population and people with migraine. Many other studies found high levels of stigma among epileptics (14, 40–44). The current study also sought to investigate the relationship between discrimination and depression, anxiety, and quality of life. Our research showed statistically significant links between discrimination and depression, anxiety, and quality of life. Previous literature also supports this notion that epilepsy is also related with a significant level of feeling of stigmatization (45), psychosocial burden (46, 47), feeling more socially restricted, lower self-efficacy (45), and somatic problems. The present study also looked at demographic differences and found non-significant differences in all demographic variables, with one exception: quality of life varies significantly by socioeconomic status, indicating that epileptic patients with high socioeconomic status have a higher level of quality of life than the other groups. Though certain psychosocial factors have been found to be linked with higher levels of reported stigma which entails low feelings about life and perceived impact of epilepsy, patient satisfaction, perceived helplessness (45), depression, anxiety, somatic symptoms and poor quality of life (47) still it was observed that patients with high level of socio economic status enjoyed good quality of life due to ease/accessto medical facility and their family awareness and conscious efforts for making their loved ones life better.

## Conclusions and implication

The present study shows that individuals with epilepsy who confront discrimination and stigma have high levels of depression and anxiety, resulting in a low quality of life. The study's findings can help us better understand the psycho-social issues that people with epilepsy face. Results of study also supports the need to consider the psycho-social issues primarily besides those related to epilepsy, its medication and use of psychological interventions as an adjunctive treatment for epileptics for enhancing their quality of life at large. The role of health care systems especially the health care providers i.e., clinical psychologists, psychiatrists, general practitioners, and medical specialists who treat epileptic patients is crucial that they must familiarize themselves with the social and cultural aspects of epilepsy, as well as close pay attention to addressing stigma in this patient population. Furthermore, future research in the field must aimed at evaluating and overcoming main factors contributing to the stigmatization process by improving the knowledge on the epilepsy through awareness culture in community and educational interventions.

### Limitations and suggestions

Results of present study should be seen cautiously because of certain limitations. Data was collected through self-report measure that may inflate the results, whereas the use of co-relational research method is another caution while considering the findings to generalize. Use of multi-method approach, combination of interview and quantitative data, or to use experimental design with control group is suggested for future research endeavors in the same domain. Finally a larger sample may be effective to examine the smaller effect size as well.

## Data availability statement

The original contributions presented in the study are included in the article/supplementary material, further inquiries can be directed to the corresponding authors.

## Ethics statement

The studies involving human participants were reviewed and approved by the IRB of the University of Sargodha, Pakistan. The patients/participants provided their written informed consent to participate in this study.

## Author contributions

NM, RF, and IU has conceptualized the idea, reviewed the literature, and collected the data. IU and AA has built the rationale, designed methodology, and carried out formal analyses. AN and SA critically evaluated, reviewed, and updated the paper. All authors also equally contributed to the final write-up of the manuscript, draft review, editing, and approval of the final manuscript. All authors contributed to the article and approved the submitted version.

## Funding

The publication of this paper was funded by Qatar National Library.

## Conflict of interest

The authors declare that the research was conducted in the absence of any commercial or financial relationships that could be construed as a potential conflict of interest.

## Publisher's note

All claims expressed in this article are solely those of the authors and do not necessarily represent those of their affiliated organizations, or those of the publisher, the editors and the reviewers. Any product that may be evaluated in this article, or claim that may be made by its manufacturer, is not guaranteed or endorsed by the publisher.

## References

[1] Boling W Means M Fletcher A. Quality of life and stigma in epilepsy, perspectives from selected regions of Asia and Sub-Saharan Africa. Brain Sci. (2018) 8:59. 10.3390/brainsci8040059 29614761 PMC5924395

[2] Lee GH Lee SA No SK Lee SM Ryu JY Jo KD Kwon JH. Factors contributing to the development of perceived stigma in people with newly diagnosed epilepsy: a one-year longitudinal study. Epilepsy Behav. (2016) 54:1–6. 10.1016/j.yebeh.2015.10.024 26610094

[3] Ranjan LK Gupta PR Srivastava M. Perceived stigma and its association with stress, anxiety, and depression among patients with epilepsy. J Nerv Ment Dis. (2022) 210:219–22. 10.1097/NMD.0000000000001431 34690275

[4] Bifftu BB Dachew BA Tiruneh BT. Perceived stigma and associated factors among people with epilepsy at Gondar University Hospital, Northwest Ethiopia: a cross-sectional institution based study. Afr Health Sci. (2015) 15:1211–9. 10.4314/ahs.v15i4.21 26958023 PMC4765415

[5] de Souza JL Faiola AS Miziara CS de Manreza ML. The perceived social stigma of people with epilepsy with regard to the question of employability. Neurol Res Int. (2018) 2018:4140508. 10.1155/2018/4140508 29862075 PMC5971250

[6] Holmes E Bourke S Plumpton C. Attitudes towards epilepsy in the UK population: results from a 2018 national survey. Seizure. (2019) 65:12–9. 10.1016/j.seizure.2018.12.012 30594807

[7] Doganavsargil-Baysal O Cinemre B Senol Y Barcin E Gokmen Z. Epilepsy and stigmatization in Turkey. Epilepsy Behav. (2017) 73:100–5. 10.1016/j.yebeh.2017.05.015 28623751

[8] Wang HJ Tan G Deng Y He J He YJ Zhou D. Prevalence and risk factors of depression and anxiety among patients with convulsive epilepsy in rural West China. Acta NeurologicaScandinavica. (2018) 138:541–7. 10.1111/ane.13016 30125939

[9] Yildirim Z Ertem DH Dirican AC Baybaş S. Stigma accounts for depression in patients with epilepsy. Epilepsy Behav. (2018) 78:1–6. 10.1016/j.yebeh.2017.10.030 29161628

[10] Vaingankar JA Chong SA Abdin E Siva Kumar FD Chua BY Sambasivam R. Understanding the relationships between mental disorders, self-reported health outcomes and positive mental health: findings from a national survey. Health Qual Life Outcomes. (2020) 18:1–0. 10.1186/s12955-020-01308-0 32131837 PMC7057535

[11] Walsh S Levita L Reuber M. Comorbid depression and associated factors in PNES vs. epilepsy: systematic review and meta-analysis. Seizure. (2018) 60:44–56. 10.1016/j.seizure.2018.05.014 29906707

[12] Oluwole LO Obadeji A Dada UM. Burden of stigma among relatives of Nigerian patients living with epilepsy. J Health Res Rev. (2015) 2:61. 10.4103/2394-2010.160913

[13] Błaszczyk B Czuczwar SJ. Epilepsy coexisting with depression. Pharmacol Rep. (2016) 68:1084–92. 10.1016/j.pharep.2016.06.011 27634589

[14] Luna J Nizard M Becker D Gerard D Cruz A Ratsimbazafy V. Epilepsy-associated levels of perceived stigma, their associations with treatment, and related factors: a cross-sectional study in urban and rural areas in Ecuador. Epilepsy Behav. (2017) 68:71–7. 10.1016/j.yebeh.2016.12.026 28109993

[15] Hopker CD Berberian AP Massi G Willig MH Tonocchi R. The individual with epilepsy: perceptions about the disease and implications on quality of life. in Codas 2017 Mar 9 (Vol. 29). SociedadeBrasileira de Fonoaudiologia. 10.1590/2317-1782/20172015236 28300952

[16] Achuko O Walker RJ Campbell JA Dawson AZ Egede LE. Pathways between discrimination and quality of life in patients with type 2 diabetes. Diabetes Technol Ther. (2016) 18:151–8. 10.1089/dia.2015.0305 26866351 PMC4790216

[17] Yeni K Tulek Z Simsek OF Bebek N. Relationships between knowledge, attitudes, stigma, anxiety and depression, and quality of life in epilepsy: a structural equation modeling. Epilepsy Behav. (2018) 85:212–7. 10.1016/j.yebeh.2018.06.019 30032810

[18] Siddiqui F Sultan T Mustafa S Siddiqui S Ali S Malik A. Epilepsy in Pakistan: national guidelines for clinicians. Pakistan J Neurol Sci. (2015) 10:47–62. Available online at: https://ecommons.aku.edu/pjns/vol10/iss3/11

[19] Khan N Jehan B Khan A Khan H. Audit of 100 cases of epilepsy in a tertiary care hospital. Gomal J Med Sci. (2011) 9:42–5. Available online at: http://www.gjms.com.pk/index.php/journal/article/view/224

[20] Asghar MA Rehman AA Raza ML Shafiq Y Asghar MA. Analysis of treatment adherence and cost among patients with epilepsy: a four-year retrospective cohort study in Pakistan. BMC Health Serv Res. (2021) 21:1–8. 10.1186/s12913-021-06085-0 33468110 PMC7816349

[21] Galateau-Salle F Dacic S Ordonez NG Churg A Hammar S. Epithelioid mesothelioma. WHO classification of tumours of the lung, pleura, thymus and heart. Lyon. (2015) 4:156–164.

[22] Eltorky M. Diffuse Malignant Mesothelioma. New York: Springer. (2015) 4:69–91. 10.1007/978-1-4939-2374-8_4

[23] Yap TA Aerts JG Popat S Fennell DA. Novel insights into mesothelioma biology and implications for therapy. Nat Rev Cancer. (2017) 17:475–88. 10.1038/nrc.2017.42 28740119

[24] Bilal A Ansari MS. Prevalence and severity of epilepsy in district Chiniot, Pakistan. Occup Med Health Affairs. (2021) 9:3. Available online at: https://www.researchgate.net/profile/Asif-Bilal-3/publication/358802340_Prevalence_and_Severity_of_Epilepsy_in_District_Chiniot_Pakistan/links/6217988f1ca59b1d50533086/Prevalence-and-Severity-of-Epilepsy-in-District-Chiniot-Pakistan.pdf

[25] Bhesania NH Rehman A Savul IS Zehra N. Knowledge, attitude and practices of school teachers towards epileptic school children in Karachi, Pakistan. Pakistan J Medical Sci. (2014) 30:220. 10.12669/pjms.301.4307 24639865 PMC3955576

[26] Ullah S Ali N Khan A Ali S Nazish HR. The epidemiological characteristics of epilepsy in the province of Khyber Pakhtunkhwa, Pakistan. Front Neurol. (2018) 9:845. 10.3389/fneur.2018.00845 30459698 PMC6232227

[27] Trinka E Kwan P Lee B Dash A. Epilepsy in Asia: disease burden, management barriers, and challenges. Epilepsia. (2019) 60:7–21. 10.1111/epi.14458 29953579

[28] Mula M Sander JW. Psychosocial aspects of epilepsy: a wider approach. BJPsych open. (2016) 2:270–4. 10.1192/bjpo.bp.115.002345 27703786 PMC4995176

[29] Asadi-Pooya AA Kanemoto K Kwon OY Taniguchi G Dong Z Chinvarun Y. Depression in people with epilepsy: How much do Asian colleagues acknowledge it? Seizure. (2018) 57:45–9. 10.1016/j.seizure.2018.03.012 29562209

[30] Lim KS Chia ZJ Myint MZ Ara KJ Chee YC Heng WT. Epilepsy in Southeast Asia, how much have we closed the management gap in past two decades? Neurol Asia. (2020) 25:425–38. Available online at: https://www.neurology-asia.org/articles/neuroasia-2020-25(4)-425.pdf

[31] Lai ST Tan WY Wo MC Lim KS Ahmad SB Tan CT. Burden in caregivers of adults with epilepsy in Asian families. Seizure. (2019) 71:132–9. 10.1016/j.seizure.2019.07.008 31325820

[32] Sherin A Gul F. Issues related to women with epilepsy in low-and middle-income countries. Khyber Med Univ J. (2017) 9:53–4.

[33] Fatima R Malik NI Jamil F Awan A Atta M. Outcomes of perceived stigma among epilepsy patients. Ann Punjab Med College. (2018) 12:138–41.

[34] Singh P Pandey AK. Quality of life in epilepsy. Int J Res Med Sci. (2017) 5:452–5. 10.18203/2320-6012.ijrms20170024

[35] King M Dinos S Shaw J Watson R Stevens S Passetti F. The Stigma Scale: development of a standardised measure of the stigma of mental illness. Br J Psychiatry. (2007) 190:248–54. 10.1192/bjp.bp.106.024638 17329746

[36] Lovibond PF Lovibond SH. The structure of negative emotional states: comparison of the depression anxiety stress scales (DASS) with the beck depression and anxiety inventories. Behav Res Ther. (1995) 33:335–43. 10.1016/0005-7967(94)00075-U 7726811

[37] Cramer JA Perrine K Devinsky O Bryant-Comstock L Meador K Hermann B. Development and cross-cultural translations of a 31-item quality of life in epilepsy inventory. Epilepsia. (1998) 39:81–8. 10.1111/j.1528-1157.1998.tb01278.x 9578017

[38] WHO. Epilepsy: Key Facts. (2022). Available online at: https://www.who.int/news-room/fact-sheets/detail/epilepsy (accessed March 16, 2022).

[39] Benson A O'Toole S Lambert V Gallagher P Shahwan A Austin JK. The stigma experiences and perceptions of families living with epilepsy: implications for epilepsy-related communication within and external to the family unit. Patient Educ Couns. (2016) 99:1473–81. 10.1016/j.pec.2016.06.009 27427482

[40] Mendes TP Crespo CA Austin JK. Family cohesion, stigma, and quality of life in dyads of children with epilepsy and their parents. J Pediatr Psychol. (2017) 42:689–99. 10.1093/jpepsy/jsw105 28137993

[41] Tareke M Birehanu M Amare D Abate A. Common mental illness among epilepsy patients in Bahir Dar city, Ethiopia: a cross-sectional study. PLoS ONE. (2020) 15:e0227854. 10.1371/journal.pone.0227854 31971965 PMC6977727

[42] Engidaw NA Bacha L Kenea A. Prevalence of depression and associated factors among epileptic patients at Ilu Ababore zone hospitals, South West Ethiopia, 2017: a cross-sectional study. Ann Gen Psychiatry. (2020) 19:1–8. 10.1186/s12991-020-00268-5 32174994 PMC7065310

[43] Shah SH Aalia B Raza MA Najeeb S Gillani S Aitazaz F. Demographic and clinical features of childhood idiopathic epilepsy at tertiary care hospital of Pakistan. Pakistan J Physiol. (2021) 17:45–9. Available online at: https://pjp.pps.org.pk/index.php/PJP/article/view/1320

[44] Tombini M Assenza G Quintiliani L Ricci L Lanzone J De Mojà R. Epilepsy-associated stigma from the perspective of people with epilepsy and the community in Italy. Epilepsy Behav. (2019) 98:66–72. 10.1016/j.yebeh.2019.06.026 31299536

[45] Ramaratnam S Baker GA Goldstein LH. Psychological treatments for epilepsy. Cochrane Database of Systematic Reviews. (2008). 10.1002/14651858.CD002029.pub3 16235293

[46] Tombini M Assenza G Quintiliani L Ricci L Lanzone J. Di Lazzaro V. Epilepsy and quality of life: what does really matter? Neurol Sci. (2021) 42:3757–65. 10.1007/s10072-020-04990-6 33449244

[47] Arnston P. The perceived psychosocial consequences of having epilepsy. Psychopathol Epilepsy Social Dimensions. (1986).

